# Rapid and accurate detection method for bluetongue virus based on CRISPR-Cas13a combined with RT-ERA

**DOI:** 10.3389/fcimb.2025.1621012

**Published:** 2025-09-01

**Authors:** Dong Zhou, Haibo Yu, Yuntong Shao, Caixia Gao, Changyou Xia, Yinglin Qi

**Affiliations:** ^1^ State Key Laboratory for Animal Disease Control and Prevention, Harbin Veterinary Research Institute, Chinese Academy of Agricultural Sciences, Harbin, China; ^2^ Heilongjiang Provincial Key Laboratory of Laboratory Animal and Comparative Medicine, Harbin Veterinary Research Institute, Chinese Academy of Agricultural Sciences, Harbin, China; ^3^ National Poultry Laboratory Animal Resource Center, Harbin Veterinary Research Institute, Chinese Academy of Agricultural Sciences, Harbin, China

**Keywords:** bluetongue virus, CRISPR-cas13a, RT-ERA, on-site detection, visualization

## Abstract

**Introduction:**

Bluetongue virus (BTV), a vector-borne pathogen of domestic and wild ruminants, poses substantial global threats to livestock health and trade. Conventional detection methods, such as RT-qPCR, remain constrained by reliance on specialized equipment and prolonged turnaround times, limiting their utility in field settings.

**Methods:**

To overcome these challenges, we developed an integrated isothermal amplification-CRISPR detection platform—Reverse Transcription-Enzymatic Recombinase Amplification coupled with CRISPR-Cas13a (RT-ERA/CRISPR-Cas13a)—enabling rapid, sensitive, specific and visual pan-serotype detection of BTV.

**Results:**

The assay demonstrated a sensitivity of 20 RNA copies/reaction within 55 min using three readout modalities: fluorescence values, visual fluorescence signals, and lateral flow test strips. Specificity evaluation revealed no cross-reactivity with 9 non-target pathogens, including epidemiologically significant viruses such as EHDV, AKAV, and CHUV. Clinical validation using 263 field samples demonstrated that RT-ERA/CRISPR-Cas13a achieved clinical sensitivities of 100%, 100%, and 96% with fluorescence values, fluorescence signals, and lateral flow strips, respectively, while maintaining 100% clinical specificity via all modalities. Field adaptation using Nucleic Acid Release Reagent (NARR) simplified crude sample processing, delivering 97% clinical sensitivity and 100% clinical specificity even in the presence of inhibitors from unpurified samples.

**Conclusion:**

This work represents the first CRISPR-Cas13a-based platform for pan-serotype BTV detection, combining portability, cost-efficiency, and detective accuracy suitable for point-of-care deployments. By bridging the gap between high laboratory sensitivity and practical field applicability, this system enables real-time BTV surveillance and facilitates timely outbreak containment in resource-constrained agricultural and veterinary settings.

## 1 Introduction

Bluetongue (BT) is an economically significant, infectious, non-contagious vector-borne viral disease that affects both domestic and wild ruminants. The disease is caused by the bluetongue virus (BTV), a member of the genus *Orbivirus* (family *Reoviridae*), and is primarily transmitted by hematophagous *Culicoides* midges (Coetzee et al., 2012). BTV possesses a segmented genome consisting of 10 linear double-stranded RNA segments, a feature that facilitates genetic reassortment and antigenic diversity. To date, 36 distinct BTV serotypes have been identified; among these, BTV-1 to BTV-27 are officially recognized by the World Organization for Animal Health (WOAH), while BTV-28 to BTV-36 are considered novel putative serotypes (Daif et al., 2022). BTV is widely distributed and has been recorded on all continents except Antarctica (Maclachlan, 2011). Its historical geographic range is between latitudes 50°N and 35°S, which aligns with the ecological distribution of the vector and temperatures required for viral replication. Since the initial introduction of BTV into Europe, the continent has experienced frequent outbreaks of BT. The most recent outbreak began in 2023 with the emergence of BTV-3, and by December 2024 a total of 42,929 cases had been reported across European countries, resulting in significant losses. Although China has not experienced an outbreak of BT since the isolation of BTV in 1979, BTV-1~5, -7, -9, -11, -12, -14~17, -20, -21, -24, and 29 have been isolated and positive antibodies have been detected in China (Zhu et al., 2013; Yang et al., 2016, Yang et al., 2017; Liao et al., 2018; Qin et al., 2018; Qi et al., 2022). This indicates that there is a risk of further outbreaks of BTV. Therefore, it is essential to establish effective point-of-care detection methods to prevent and control BTV outbreaks.

Although several laboratory-based diagnostic methods for BTV detection exist—including virus isolation, serological assays, and nucleic acid amplification techniques—their operational constraints restrict applicability in field settings (Rojas et al., 2019). Virus isolation, historically regarded as the gold standard, is confined to biosafety level 3 (BSL-3) containment facilities due to stringent biocontainment requirements. Serological methods, particularly enzyme-linked immunosorbent assays (ELISAs), serve as the cornerstone for population-level serosurveillance but lack diagnostic utility during the critical 7-day post-infection window preceding detectable antibody seroconversion (Hayrapetyan et al., 2023). Molecular diagnostics such as reverse transcription PCR (RT-PCR) and quantitative RT-PCR (RT-qPCR) achieve superior sensitivity, yet their requirement for thermocycling equipment, operator expertise, and extended processing times (4–6 hours) preclude field deployment. Collectively, these constraints highlight the urgent demand for a novel diagnostic method capable of rapid, specialized equipment-free, on-site BTV detection with high sensitivity and specificity.

Recent advances in CRISPR-based diagnostics (CRISPR-Dx) have revolutionized pathogen detection through programmable nucleic acid recognition. These systems employ Cas effector proteins (e.g., Cas12, Cas13) complexed with guide crRNAs to specifically identify target sequences, subsequently activating their collateral cleavage activity that nonspecifically degrades reporter molecules (Zetsche et al., 2015; East-Seletsky et al., 2016). For optimal sensitivity, CRISPR-Dx is typically coupled with isothermal amplification methods. Integration with isothermal amplification techniques—such as Reverse Transcription Recombinase Polymerase Amplification (RT-RPA) and Reverse Transcription Loop-mediated Isothermal Amplification (RT-LAMP), and PCR has led to the development of innovative platforms like DETECTR, SHERLOCK, and HOLMES (Gootenberg et al., 2017; Chen et al., 2018; Li et al., 2019). These platforms have a significant advantage, as they enable on-site visual detection of pathogens without requiring specialized equipment, all while maintaining high sensitivity and specificity. For example, Zhang et al. established the RPA-LwCas13a-LFS method for visually detecting African Swine Fever Virus (ASFV), achieving a sensitivity level as low as 2 copies/μL (Zhang et al., 2024). In addition, Reverse Transcription-Enzymatic Recombinase Amplification (RT-ERA) integrates reverse transcriptase with recombinase-primer complexes, enabling rapid RNA detection under isothermal conditions for downstream detection (Piepenburg et al., 2006; Xia and Chen, 2020).

The field of molecular diagnostics has witnessed rapid advancements not only in CRISPR-Cas detection methodologies but also in parallel developments of rapid nucleic acid release techniques. Cameron Myhrvold et al. developed Heating Unextracted Diagnostic Samples to Obliterate Nucleases (HUDSON), a groundbreaking approach for field-deployable viral diagnostics that revolutionized sample preparation through two key mechanisms: (1) TCEP-mediated cleavage of protein disulfide bonds to enhance lysis efficiency, and (2) EDTA chelation of Mg²^+^/Ca²^+^ to inhibit residual nuclease activity (Myhrvold et al., 2018). Building upon this foundation, GenDx (China) have developed a next-generation Nucleic Acid Release Reagent (NARR, CST0016) for rapid nucleic acid extraction employing amplification-compatible chemical formulations combined with highly efficient surfactants to achieve rapid, robust cellular lysis while maintaining compatibility with downstream molecular assays. These advancements establish CRISPR-Dx as a powerful and versatile tool for diagnostics across diverse pathogens.

**Figure 1:**
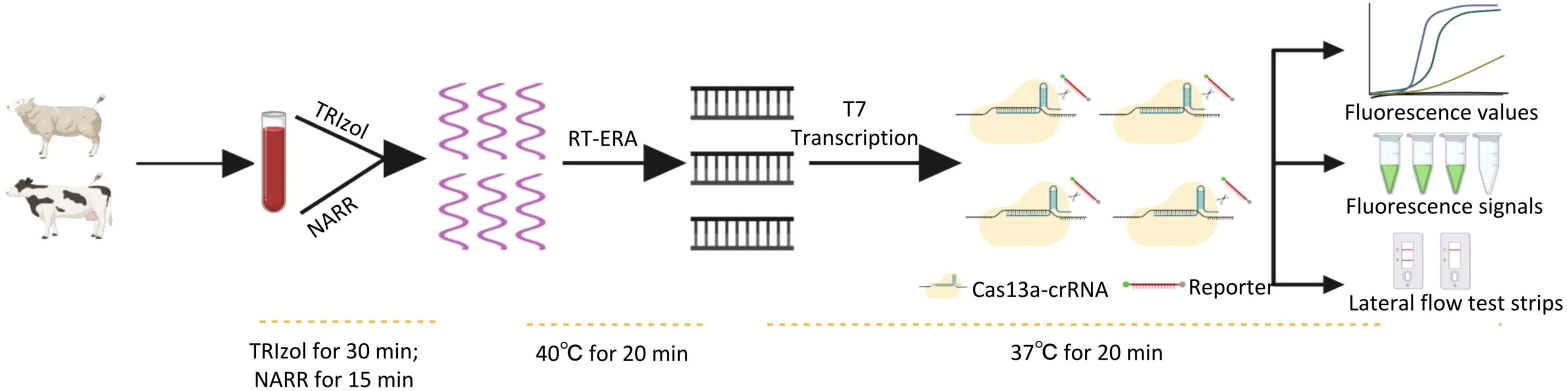
Schematic workflow for detection based on RT-ERA/CRISPR-Cas13a.

Therefore, in this study, we developed a novel CRISPR-Cas13a-based diagnostic method capable of rapidly and accurately detecting BTV with sensitivity down to 20 RNA copies/reaction. This system integrates RT-ERA with CRISPR-Cas13a collateral cleavage activity, enabling multimodal readouts via fluorescence values, fluorescence signal visualization, and lateral flow test strips. Furthermore, the incorporated NARR streamlines field-compatible sample processing, allowing crude lysate-to-result detection within 55 min without specialized equipment (**Figure 1**). This innovation provides a robust, adaptable, and cost-effective solution for BTV monitoring in resource-constrained ruminant production systems.

## 2 Materials and methods

### 2.1 Virus and sample collection

The inactivated virus and samples of blood and tissue homogenates from bovine and ovine were obtained from the Livestock Infectious Diseases Research Team at Harbin Veterinary Institute, Chinese Academy of Agricultural Sciences. The Animal Ethics Committee of Harbin Veterinary Research Institute did not require ethical review or approval for this study.

The inactivated viruses include various strains of BTV (BTV-1, -2, -4, -7, -9, -12, -15, -16, -20, and -21), Epidemic hemorrhagic disease virus (EHDV), Akabane disease virus (AKAV), Chuzan virus (CHUV), Peste des petits ruminants virus (PPRV), Goat Pox virus (GPV), Bovine respiratory syncytial virus (BRSV), Bovine rotavirus (BRV), Bovine parainfluenza virus type 3 (BPIV3), and Infectious bovine rhinotracheitis virus (IBRV).

The segment 1 (S1) sequence of BTV, containing the T7 promoter, was cloned from multiple BTV serotypes (BTV-3, -5, -6, -8, -10, -11, -13, -14, -17, -18, -19, -22, -23, -24, -25, -27, -28, -29, -32 and -33) (**Supplementary Table 1**) into the pUC57 vector and subsequently transcribed *in vitro* using the T7 High Yield RNA Transcription Kit (Vazyme, China). The RNA product was purified via a GeneJET RNA Purification Kit (Thermo Fisher Scientific, USA) and stored at -80°C.

### 2.2 CrRNA sequence and RT-ERA primer design

BTV is classified into 36 serotypes. For this study, the sequences of 30 serotypes of BTV were obtained from GenBank (**Supplementary Table 1**) and aligned using MEGA 11. Regarding the remaining 6 serotypes: BTV-26 and -31 were intentionally excluded due to excessive sequence divergence in the crRNA target regions, while complete genomic sequences for BTV-30, -34, -35, and -36 were unavailable in GenBank. Through this analysis, we identified highly conserved regions within S1, S8, S9, and S10 as optimal targets for detection. Leveraging the protospacer flanking sequence (PFS) requirements and the known tolerance of the CRISPR-Cas system to single-base mismatches in crRNA recognition, we designed multiplex detection assays targeting these conserved regions. Sequence analysis revealed that all 30 BTV serotypes either perfectly matched with our designed crRNA or contained only single-nucleotide mismatches. The complete crRNA sequences (**Table 1**) were commercially synthesized by GenScript Biotech Co., Ltd.

**Table 1:** CrRNA sequence information designed for different segments of the BTV genome.

Segment targets	crRNA names	crRNA sequence bases (5’-3’)
S1	crRNA1	GAUUUAGACUACCCCAAAAACGAAGGGGACUAAAACGGAUAAAAGCGUUCGACCACUCGCUUGA
	crRNA2	GAUUUAGACUACCCCAAAAACGAAGGGGACUAAAACGGAUAAAACCGUUCAACCACUCGCUUGA
	crRNA3	GAUUUAGACUACCCCAAAAACGAAGGGGACUAAAACGGAUAAAAGCGCUCGACCACCCGUUUGA
S8	crRNA1	GAUUUAGACUACCCCAAAAACGAAGGGGACUAAAACUAAACGCCGACCGGCAAUAUGAUCAAGC
	crRNA2	GAUUUAGACUACCCCAAAAACGAAGGGGACUAAAACUAAACACCGACCGGCAAUAUGAUUAAGC
S9	crRNA1	GAUUUAGACUACCCCAAAAACGAAGGGGACUAAAACACCUCCUCCUCCAACCUUUCCAUCUCCU
	crRNA2	GAUUUAGACUACCCCAAAAACGAAGGGGACUAAAACACCUCCUCCUCUAGUCUUUCCAUCUCCU
S10	crRNA1	GAUUUAGACUACCCCAAAAACGAAGGGGACUAAAACUAGCACCCGUUGUAUUUGACAUCGCUUU
	crRNA2	GAUUUAGACUACCCCAAAAACGAAGGGGACUAAAACUUGCACCAGUAGUGUUUGACAUCGCUUU

Following crRNA mixture screening, we selected target segments from S1, S8, S9, and S10 for primer development. According to the primer design principles, we designed six forward primers (F1-F6, **Table 2**) and five reverse primers (R1-R5, **Table 2**), all of which were commercially synthesized by Ruibo Biotech Co., Ltd.

**Table 2:** RT-ERA primers designed for BTV genomic segment 1.

Primers name	Base sequence (5’-3’)
F1	TAATACGACTCACTATAGGGTGCAATGGTCGCAATYACCGTGCARGGTGC
F2	TAATACGACTCACTATAGGGAATGCAATGGTCGCAATYACCGTGCARG
F3	TAATACGACTCACTATAGGGAATGCAATGGTCGCAATYACCGTGCAR
F4	TAATACGACTCACTATAGGGATGCAATGGTCGCAATYACCGTGCAR
F5	TAATACGACTCACTATAGGGTGCAATGGTCGCAATYACCGTGCAR
F6	TAATACGACTCACTATAGGGAATGGTCGCAATYACCGTGCARGGTGCACAGC
R1	GRTAYTTHGTWCCATGYTTCATYCTWAT
R2	GRTAYTTHGTWCCATGYTTCATYCTWA
R3	GRTAYTTHGTWCCATGYTTCATYCTW
R4	YYGRTAYTTHGTWCCATGYTTCATYC
R5	GARAACTTRTATATRTARCATGCYCCTTC

### 2.3 RT-ERA

The RT-ERA (GenDx, China) reaction consisted of a total volume of 10 μL, containing Reaction buffer (4 μL), Forward primer (0.5 μL of 10 μM stock), Reverse primer (0.5 μL of 10 μM stock), Template (1 μL), Activator (0.4 μL), RNase-free water (to a final volume of 10 μL). The reaction was incubated at 40°C for 20 min.

### 2.4 CRISPR-Cas13a

The CRISPR-Cas13a reaction consisted of a total volume of 10 μL, containing Cas13a protein, crRNA mixture, a single-stranded RNA fluorescence reporter (Fam-UUUUUU-BHQ-I) or a biotin-labeled single-stranded RNA probe (FAM-UUUUUU-Biotin) (1 μL of 10 μM stock), recombinant RNase inhibitor (RRI, 0.4 μL, Takara, Japan), 10 × Cas13a buffer (1 μL, GenScript, USA), ATP/GTP/CTP/UTP (0.5 µL each of 5 mM stock, Vazyme, China), T7 RNA polymerase mix (1 µL, Vazyme, China), 10 × T7 reaction buffer (1 µL, Vazyme, China), RT–ERA product (1 μL), and RNase-free water (to a final volume of 10 μL).

In this study, three approaches were employed to analyze CRISPR-Cas13a detection results: (1) Fluorescence values: CRISPR-Cas13a were performed on the QuantStudio™ 5 Real-Time PCR System (Thermo Fisher Scientific, USA) under isothermal conditions (37°C for 20 min), with fluorescence values recorded at 30-second intervals. (2) Fluorescence Signals: following the completion of the CRISPR-Cas13a reaction (incubated at 37°C for 20 minutes in a QuantStudio™ 5 Real-Time PCR System or thermostat water bath), fluorescence signals were visually captured using a blue light imaging system. (3) Lateral flow strip test: detection was performed using CRISPR-specific Lateral Flow Test Strips (GenDx, China) for rapid visual interpretation.

### 2.5 Optimization of the RT–ERA/CRISPR-Cas13a detection system

The detection efficiency of the RT-ERA/CRISPR-Cas13a system is influenced by multiple interdependent factors. To maximize sensitivity and specificity, we systematically optimized three critical parameters, including crRNA mixture composition, Cas13a protein (GenScript, USA) and crRNA concentrations, and RT-ERA primer selection. For crRNA screening, we designed mixtures targeting conserved regions in S1, S8, S9, and S10, which were evaluated against four representative BTV serotypes (20 ng RNA of BTV-1, -15, -16, and -20).

For Cas13a protein and crRNA concentrations, a systematic optimization was performed by first testing a concentration gradient of Cas13a protein (50, 75, 100, and 200 ng per 10 μL reaction) in the CRISPR-Cas13a system. Following identification of the optimal Cas13a concentration, we titrated crRNA concentrations across a six-point gradient (0.1, 0.2, 0.5, 1, 2, and 4 μM) to determine the minimal effective dose. During system optimization, the *in vitro*-transcribed BTV S1 RNA was maintained at 2 × 10¹¹ copies/reaction. The quantity of the initial RNA template was calculated using the following formula: Amount (copies/μL) = [RNA concentration (g/μL)/(bp × 340)] × 6.02 × 10²³.The final reaction mixture consisted of T7-transcribed target RNA, optimized buffer, and RNase inhibitor to ensure RNA stability.

For the selection of RT-ERA primers, a sequential screening strategy was employed, wherein primer pairs were cross-validated using CRISPR-Cas13a-mediated fluorescence detection. For the RT-ERA reaction, RNA templates were used at 2 × 10^5^ copies/reaction, and the amplification products were utilized as substrates for CRISPR-Cas13a analysis. Initial screening fixed reverse primer R3 while testing forward primers F1-F6, followed by evaluation of reverse primers R1-R5 using the optimal forward primer. Primer pairs were ranked based on fluorescence values and reaction kinetics, and the most suitable pair was selected for further applications.

### 2.6 Evaluation of the RT–ERA/CRISPR-Cas13a detection system

We evaluated the analytical sensitivity of the RT-ERA/CRISPR-Cas13a system using serial dilutions of *in vitro*-transcribed BTV S1 RNA (2000, 200, 20, 2 copies/reaction). The limit of detection (LOD) for the RT-ERA/CRISPR-Cas13a was determined through 20 independent replicate experiments.

The analytical specificity was validated against arthropod-borne viruses: EHDV, AKAV, and CHUV; and common bovine/ovine pathogens: PPRV, GPV, BRSV, BRV, BPIV3, and IBRV. All viral strains were inactivated, cell-culture-derived preparations, and detection was performed using 20 ng RNA.

Through screening of segment-targeting crRNA mixture, we optimized a triplex crRNA mixture (crRNA1-3) targeting conserved S1 regions. Bioinformatic alignment verified theoretical recognition of all 30 BTV serotypes. Experimental validation comprised: (i) testing with available viral strains (BTV-1, -2, -4, -7, -9, -12, -15, -16, -20, and -21), and (ii) evaluation of synthetic S1 RNAs for unavailable serotypes (BTV-3, -5, -6, -8, -10, -11, -13, -14, -17, -18, -19, -22, -23, -24, -25, -27, -28, -29, -32, and -33), which were produced via T7 polymerase-mediated *in vitro* transcription. Sequence analysis identified single-nucleotide mismatches between the crRNA cocktail and BTV-6, -14, -25, -28, -29, and -33, all of which were included in synthetic validation. This comprehensive assessment of all targeted serotypes conclusively demonstrates the pan-serotypic detection capability of our RT-ERA/CRISPR-Cas13a platform. The sensitivity and specificity were validated through three modalities: fluorescence values, fluorescence signals, and lateral flow test strips. And the pan-serotype detection capability was confirmed by fluorescence values.

### 2.7 Detection of clinical samples

We set the clinical sensitivity and specificity at 95% with a 95% confidence interval (CI) and a margin of error (Δ) of ± 5%. Based on statistical calculations requiring a minimum of 73 positive and 73 negative samples, we ultimately selected 80 positive and 183 negative samples, meeting statistical requirements. Parallel testing was conducted using: (1) the reference method specified in China’s National Standard GB/T 18636-2017 “Diagnostic Techniques for Bluetongue Virus” (promulgated by the Standardization Administration of China and Ministry of Agriculture and Rural Affairs), and (2) our novel RT-ERA/CRISPR-Cas13a platform. Comparative evaluation against RT-qPCR results demonstrated the diagnostic performance characteristics of the RT-ERA/CRISPR-Cas13a system.

For BTV nucleic acid detection in field, a NARR-based protocol was employed: 50 μL of tissue homogenate supernatant or whole blood was mixed with 450 μL of Nucleic Acid Release Reagent (GenDx, CST0016), vortexed, heat-denatured at 95°C for 5 min, and centrifuged at 3000 g for 1 min. The resulting crude lysate supernatant was then directly analyzed using the RT-ERA/CRISPR-Cas13a detection system.

### 2.8 Statistical analysis

Data analysis was performed using GraphPad Prism 10.0 software (GraphPad Software, San Diego, CA, USA), with measurement data expressed as mean ± standard deviation (mean ± SD). For diagnostic accuracy analysis, both sensitivity (true positives/[true positives + false negatives]) and specificity (true negatives/[false positives + true negatives]) were evaluated using exact binomial 95% CI (Clopper-Pearson method).

## 3 Results

### 3.1 The optimization outcomes of the RT-ERA/CRISPR-Cas13a detection system

**Figure 2:**
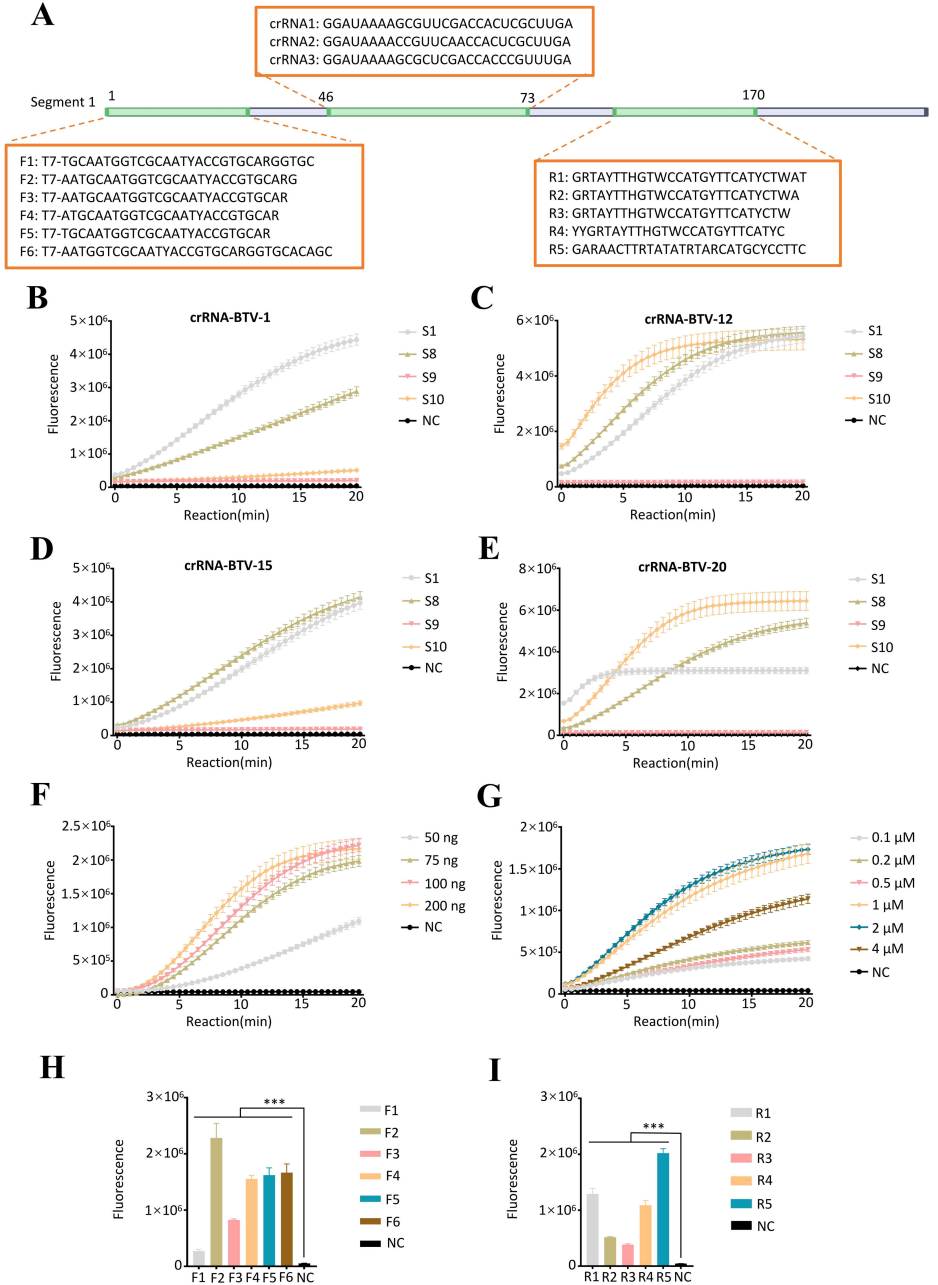
Establishment of the RT–ERA/CRISPR-Cas13a detection system. **(A)** RT-ERA primer and crRNA binding sites targeting the BTV segment 1 (S1). **(B-E)** Evaluation of crRNA combinations targeting S1/S8/S9/S10 across BTV serotypes via fluorescence values: **(B)** BTV-1, **(C)** BTV-15, **(D)** BTV-16, **(E)** BTV-20. **(F, G)** Optimization of CRISPR-Cas13a system: **(F)** Cas13a protein amount, **(G)** crRNA concentration. **(H, I)** Cross-screening of RT-ERA primers: **(H)** Upstream primer screening, **(I)** Downstream primer screening. NC, negative control of RNase-free ddH_2_O. Error bars in panels represent the mean ± SD, where n = 3 replicates. The fluorescence values of all bars are the end values of the determination. ***p<0.001.

To evaluate the detection efficiency mediated by crRNA in the CRISPR-Cas13a system, we incubated four crRNA mixtures targeting BTV S1, S8, S9, and S10 with RNA extracted from representative serotypes (BTV-1, -15, -16, and -20). As shown in **Figures 2A-E**, the real-time fluorescence values indicated that the S1-targeting crRNA mixture exhibited the highest detection efficiency. In contrast, the negative controls (RNase-free ddH_2_O) showed no response. Consequently, the S1-specific crRNA mixture was chosen for subsequent experiments.

Following crRNA mixture screening, we systematically optimized the CRISPR-Cas13a detection system by titrating key reaction components. Various concentrations of Cas13a protein (50–200 ng) and crRNA mixtures (0.1-4 μM) were evaluated, and it was determined that 75 ng of Cas 13a protein and 1 μM of crRNA mixture (crRNA1: crRNA2: crRNA3 = 1: 1: 1) (1 μL of 10 μM total, prepared from 0.33 μL each of 10 μM stocks) were the optimal conditions for the reaction (**Figures 2F, G**).

To ensure optimal detection efficiency of the RT-ERA/CRISPR-Cas13a system, we performed systematic primer screening for RT-ERA using CRISPR-Cas13a-mediated fluorescence monitoring. Forward primers (F1-F6) and reverse primers (R1-R5) targeting the S1 were cross-validated through iterative combinatorial screening, with amplification efficiency assessed by real-time fluorescence intensity. In the initial validation step, reverse primer R3 was fixed while forward primers F1-F6 were tested, revealing F2 as the highest-performing candidate. Subsequent evaluation of reverse primers R1-R5, using F2 as the anchor primer, identified R5 as the optimal reverse primer. The results indicated that the F2/R5 combination demonstrated superior amplification efficiency compared to other primer pairs (**Figures 2H, I**).

### 3.2 Evaluation of detection with RT–ERA/CRISPR-Cas13a

**Figure 3:**
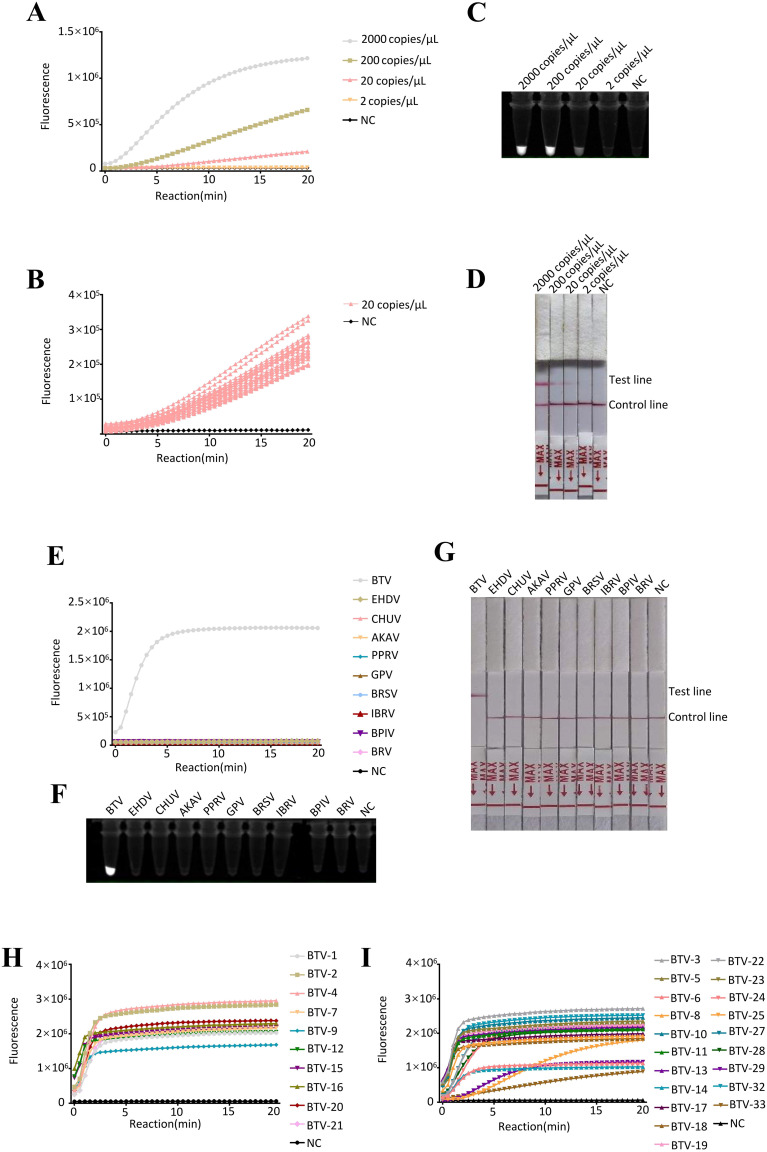
Evaluation of detection with RT–ERA/CRISPR-Cas13a. **(A-D)** Sensitivity evaluation of RT-ERA/CRISPR-Cas13a using RNA templates (2000, 200, 20, 2 copies/reaction). Detection modalities: **(A, B)** Fluorescence values, **(C)** Fluorescence signals, **(D)** Lateral flow test strip. **(E-G)** Specificity evaluation of RT-ERA/CRISPR-Cas13a against EHDV, AKAV, CHUV, PPRV, GPV, BRSV, BRV, BPIV3, and IBRV. Detection modalities: **(E)** Fluorescence values, **(F)** Fluorescence signals, **(G)** Lateral flow test strip. **(H, I)** Pan-serotype validation of RT-ERA/CRISPR-Cas13a through detection of 16 BTV serotypes. NC, negative control of RNase-free ddH_2_O.

To determine the analytical sensitivity of the RT-ERA/CRISPR-Cas13a detection system, serial dilutions of *in vitro*-transcribed BTV S1 RNA (2000, 200, 20 and 2 copies/reaction) were analyzed. The assay consistently detected as low as 20 RNA copies/reaction (**Figures 3A-D**). These results confirm the robust performance of the system in detecting low-abundance BTV RNA.

To assess the analytical specificity of the RT-ERA/CRISPR-Cas13a detection system, we evaluated cross-reactivity against vector-borne pathogens (EHDV, AKAV, and CHUV) and common bovine and ovine pathogens (PPRV, GPV, BRSV, BRV, BPIV3, and IBRV). Fluorescence values, fluorescence signals, and lateral flow test strips collectively demonstrated specific reactivity to BTV, with no detectable cross-reactivity observed for any of the non-target pathogens (**Figures 3E-G**). This confirms that the RT-ERA/CRISPR-Cas13a detection system enables highly specific detection of BTV.

The pan-serotype detection capability of the RT-ERA/CRISPR-Cas13a system was comprehensively validated through the testing of 30 BTV serotypes. Fluorescence value results demonstrated efficient detection of: (i) virus strains (BTV-1, -2, -4, -7, -9, -12, -15, -16, -20, and -21) and (ii) synthetic S1 RNAs representing unavailable serotypes (BTV-3, -5, -6, -8, -10, -11, -13, -14, -17, -18, -19, -22, -23, -24, -25, -27, -28, -29, -32, and -33) (**Figures 3H, I**). These findings conclusively establish that the RT-ERA/CRISPR-Cas13a platform can reliably detect at least 30 distinct BTV serotypes.

### 3.3 The detection results of clinical samples

**Figure 4:**
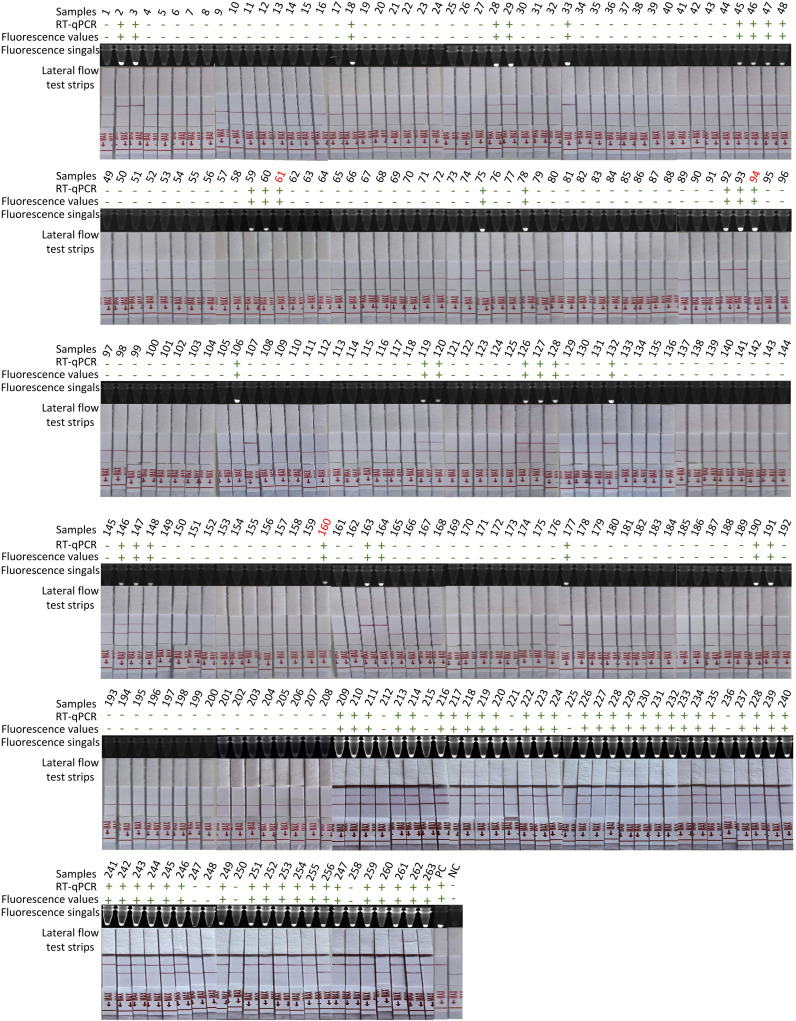
Detection of 263 clinical samples. Following RNA extraction with TRIzol, parallel testing was performed by RT-qPCR and RT-ERA/CRISPR-Cas13a through three modalities: fluorescence values, fluorescence signals, and lateral flow test strips. Samples with discordant results between methods are highlighted in red numerals. “+”: positive; “-”: negative; PC, positive control; NC, negative control of RNase-free ddH_2_O.

To validate the clinical applicability of the RT-ERA/CRISPR-Cas13a system, we analyzed RNA extracted from 263 clinical samples (80 positive and 183 negative). Following RNA extraction using TRIzol reagent, initial testing with reference RT-qPCR (GB/T 18636-2017) accurately identified 80 positive and 183 negative samples (**Table 3**, **Figure 4**), matching the clinical assay diagnosis. The RT-ERA/CRISPR-Cas13a system demonstrated perfect diagnostic concordance, detecting all 80 positives and 183 negatives through both fluorescence values and fluorescence signal, achieving 100% clinical sensitivity and specificity (**Table 3**, **Figure 4**). Lateral flow immunochromatographic analysis identified 77 true positives among the 80 confirmed cases (96% sensitivity) (**Table 3**, **Figure 4**) while correctly classifying all 183 negative samples (100% specificity) (**Table 3**, **Figure 4**). These results robustly confirm the system’s diagnostic accuracy under controlled laboratory conditions.

**Table 3:** The results of clinical sample detecting.

Treatment	Clinical sensitivity (95%CI)	Clinical specificity (95%CI)
TRIzol-RT-qPCR	100% (95%, 100%)	100% (98%, 100%)
TRIzol-RT–ERA/CRISPR-Cas13a-Fluorescence values	100% (95%, 100%)	100% (98%, 100%)
TRIzol-RT–ERA/CRISPR-Cas13a-Fluorescence signals	100% (95%, 100%)	100% (98%, 100%)
TRIzol-RT–ERA/CRISPR-Cas13a-Lateral flow test strips	96% (89%, 99%)	100% (98%, 100%)
NARR-RT–ERA/CRISPR-Cas13a-Fluorescence signals	97% (91%, 100%)	100% (98%, 100%)

To address operational constraints in resource-limited settings (including limited access to precision instrumentation and budgetary considerations), we developed a streamlined NARR-based sample processing protocol. This field-optimized workflow enabled direct analysis of crude lysates in RT-ERA reactions, eliminating nucleic acid purification requirements while maintaining analytical performance. The modified NARR/RT-ERA/CRISPR-Cas13a platform correctly identified 78 of 80 true positive samples (97% sensitivity) (**Table 3**) and all negative controls (100% specificity) (**Table 3**), demonstrating two false-negative occurrences under field-simulated conditions.

This demonstrates that the RT-ERA/CRISPR-Cas13a-based BTV detection method is capable of providing visible results without the need for sophisticated instruments, thus enabling rapid on-site screening of ruminant populations in resource-constrained field settings.

## 4 Discussion

In this study, we developed an integrated RT-ERA/CRISPR-Cas13a system that enables the rapid, sensitive, specific, visual, and specialized instrument-free detection of BTV. By integrating RT-ERA with CRISPR-Cas13a-mediated collateral cleavage activity, the system achieves multimodal readouts (fluorescence values, fluorescence signals, lateral flow test strips), effectively addressing critical gaps in current BTV diagnostics.

First, the assay demonstrated a detection limit as low as 20 RNA copies/reaction, achieving high sensitivity for low-abundance BTV RNA. This performance approaches the analytical sensitivity of the RT-qPCR assay developed by Maan et al. (LOD is 4 copies/reaction) and surpasses that reported by Mulholland et al. (LOD is 200 copies/reaction), positioning our method competitively among current BTV detection systems (Maan et al., 2015; Mulholland et al., 2017). Given the overlapping clinical manifestations caused by vector-borne viruses such as EHDV, AKAV, and CHUV (hemorrhage, edema, and mucosal damage) and the potential for co-infections in ruminants, specificity is paramount for accurate BTV diagnosis. Through three detection modalities, we confirmed that the RT-ERA/CRISPR-Cas13a system exhibits exclusive reactivity to BTV with no observed cross-reactivity to non-target pathogens. This specificity, coupled with the inherent programmability of CRISPR-Dx, enables tailored optimization for other pathogens, thereby establishing a versatile framework for developing multiplex detection systems. Building on this adaptability, we are currently engineering a CRISPR-Cas-based multiplex system that leverages the orthogonal cleavage activities of Cas proteins paired with distinct fluorescent reporter probes, inspired by the pioneering work of Gootenberg et al (Gootenberg et al., 2018). This advancement could simultaneously detect high-risk serotypes while maintaining serotype-specific resolution (Szmaragd et al., 2007; Voigt et al., 2024).

Second, our assay addresses the critical challenge of BTV serotype diversity through broad-spectrum detection capability. The extensive genetic variability of BTV, driven by host diversity, viral recombination, and environmental adaptation, has resulted in 36 recognized serotypes that complicate surveillance efforts. Our method successfully detects 10 distinct BTV serotype strains and 20 synthetic BTV segments representing additional serotypic variations, thereby exceeding the serotype coverage of currently available detection methods. While further validation with additional strains is planned to establish a more comprehensive surveillance system, the current results demonstrate superior broad-spectrum detection that will significantly enhance capabilities for tracking viral evolution, providing early outbreak warning, and implementing precision control measures.

Third, we implemented multimodal detection readouts to balance precision and field applicability. Fluorescence values measurement permits real-time reaction monitoring and precise endpoint determination, while fluorescence signals and lateral flow test strips provide rapid visualization results. Collecting fluorescence values using instruments enables the monitoring of the reaction process, and a change in fluorescence values can be interpreted as a positive result. This approach allows for continuous observation and precise measurement of the reaction, providing reliable data for analysis. In comparison, when determining positivity using fluorescence signals and test strips, there is a threshold—specifically, the product must accumulate to a certain level before it can be considered positive. Although visual detection may have a slight impact on the accuracy of reading results, a significant advantage of this method is that it eliminates the reliance on specialized equipment. This feature makes the method more accessible and user-friendly, allowing for quicker interpretation of results in various settings, including those with limited laboratory resources.

This study systematically evaluated the clinical performance of the RT-ERA/CRISPR-Cas13a detection system. Results demonstrated 100% clinical sensitivity for both fluorescence values and fluorescence signals, while lateral flow test strips showed 96% sensitivity, suggesting potential sensitivity loss in visual readouts particularly for low viral load samples. Under field conditions using the NARR method, sensitivity reached 97%, representing a marginal decrease from laboratory standards while maintaining high detection efficacy. Notably, all four detection conditions exhibited 100% clinical specificity, highlighting the exceptional target-discrimination capacity of CRISPR-Cas13a technology. Key findings indicate that: (1) fluorescence-based detection is recommended in laboratory settings for optimal sensitivity; (2) the NARR method represents a viable field-deployable alternative that preserves high specificity while improving operational efficiency; (3) further optimization of the lateral flow strips transduction system is needed to enhance sensitivity. The perfect specificity (zero false-positive rate) establishes its significant value for both clinical diagnostics and field surveillance. However, we recognize that the current sample size is inadequate for more robust statistical analyses, including power calculations and effect size estimations. We will conduct large-scale validation studies after collecting additional samples in subsequent research.

While the RT-ERA/CRISPR-Cas13a system offers significant advantages, certain limitations warrant acknowledgment. Although BTV comprises 36 recognized serotypes, our current assay demonstrated detection of 30 serotypes, leaving 6 serotypes unaddressed. This gap likely arises from sequence divergence in primer/probe binding regions, necessitating further optimization of crRNA designs or amplification conditions to expand serotype coverage. Furthermore, cost-effectiveness remains a concern for large-scale implementation. At present, the total reaction cost ($3.69 per test)—comprising RT-ERA ($1.44), CRISPR-Cas13a ($0.40), and T7 transcription ($1.85)—exceeds that of conventional RT-qPCR ($3 per test). Competitive pricing could be achieved through component streamlining, such as replacing commercial T7 polymerase with in-house expressed variants or integrating transcription-amplification steps into a single-tube workflow. Overall, although the RT-ERA/CRISPR-Cas system is 23% more expensive than RT-qPCR, the method developed in this study eliminates the need for costly precision instruments and reduces reliance on specialized personnel. However, this potential advantage requires validation through end-user testing. Additionally, it should be noted that our selected target differs from the WOAH-recommended S10 for nucleic acid detection. During our evaluation of the CRISPR-Cas13a system, targeting the S10 showed reduced detection efficiency against BTV-1 and BTV-15. We therefore opted for the S1 based on its superior detection performance across all tested serotypes. While this constitutes a departure from the WOAH standard protocol, extensive validation studies have demonstrated that S1 targeting maintains high detection efficiency, positioning it as a viable candidate for next-generation BTV detection standards. Although a multiplex approach targeting both S1 and S10 could theoretically be implemented, this would introduce substantial technical challenges in achieving consistent crRNA performance across multiple targets.

In conclusion, the RT-ERA/CRISPR-Cas13a system represents a transformative advancement in BTV detection, combining high sensitivity, stringent specificity, and field-deployable simplicity. Its ability to deliver multimodal readouts (fluorescence values, fluorescence signals, and lateral flow strips) enables adaptable deployment across diverse settings—from resource-limited farms to well-equipped laboratories. By facilitating rapid, large-scale screening, this method empowers veterinarians and farmers to implement timely containment measures, thereby mitigating outbreak risks in livestock populations. Moreover, its compatibility with crude lysates via NARR pretreatment enhances practicality for on-site testing without compromising diagnostic accuracy. Beyond clinical applications, this technology holds promise for epidemiological surveillance, enabling real-time tracking of BTV serotype distribution and evolution. The establishment of this method not only elevates detection efficiency but also contributes substantively to global One Health initiatives by safeguarding animal welfare, ensuring food security, and stabilizing the economic viability of livestock industries. Future efforts will focus on enhancing serotype inclusivity, reducing costs, and developing multiplexing capabilities to address emerging challenges in arbovirus diagnostics.

## Data Availability

The original contributions presented in the study are included in the article/**Supplementary Material**. Further inquiries can be directed to the corresponding author/.
