# Supplementary material for: Rapid and accurate detection method for bluetongue virus based on CRISPR-Cas13a combined with RT-ERA
Source: Front Cell Infect Microbiol. 2025 Sep 1;15:1621012. doi: 10.3389/fcimb.2025.1621012 (PMC12434134; doi:10.3389/fcimb.2025.1621012)
Supplement: Supplementary file 1 [file Table1.docx]

Supplementary Material

# Supplementary Figures and Tables

**Supplementary Table 1 GenBank Accession Numbers for BTV**

| Serotype | GenBank | Serotype | GenBank |
| --- | --- | --- | --- |
| BTV-1 | JX680457.1 | BTV-16 | JX129381.1 |
| BTV-2 | KP820910.1 | BTV-17 | MT952971.1 |
| BTV-3 | KP820919.1 | BTV-18 | MT078369.1 |
| BTV-4 | KP820946.1 | BTV-19 | MZ215891.1 |
| BTV-5 | MN710093.1 | BTV-20 | [OL333534.1](https://www.ncbi.nlm.nih.gov/nucleotide/OL333534.1?report=genbank&log$=nucltop&blast_rank=2&RID=UVT9JWWM013) |
| BTV-6 | GQ506498.1 | BTV-21 | MT078399.1 |
| BTV-7 | MN710094.1 | BTV-22 | OP185864.1 |
| BTV-8 | [GQ506451.1](https://www.ncbi.nlm.nih.gov/nucleotide/GQ506451.1?report=genbank&log$=nucltop&blast_rank=59&RID=UVSXSDGU016) | BTV-23 | MT090655.1 |
| BTV-9 | KP820968.1 | BTV-24 | OP185834.1 |
| BTV-10 | MN710095.1 | BTV-25 | GQ982522.1 |
| BTV-11 | MN710096.1 | BTV-27 | LN713671.1 |
| BTV-12 | MN710097.1 | BTV-28 | MH559813.1 |
| BTV-13 | MN710098.1 | BTV-29 | KX695170.1 |
| BTV-14 | KP820863.1 | BTV-32 | KX234078.3 |
| BTV-15 | KP820864.1 | BTV-33 | LR877337.1 |
